# Unusual Pattern of Osteochondral Marginal Impaction in Acetabular Fractures: A Report of Two Cases

**DOI:** 10.1055/s-0042-1755347

**Published:** 2022-09-05

**Authors:** Bahaaeldin Ibrahim, Mahmoud Badran, Ahmed A. Khalifa, Hossam Abubeih, Osama Farouk

**Affiliations:** 1 Departamento de Ortopedia, Assiut University, Assiut, Egito; 2 Departamento de Ortopedia, Al-Azhar University, Assiut, Egito; 3 Departamento de Ortopedia, Qena Faculty of Medicine and University Hospital, South Valley University, Qena, Egito

**Keywords:** acetabulum, fractures, bone, hip fractures, osteonecrosis

## Abstract

Acetabular fractures could be accompanied by articular impaction injuries, affecting the outcomes if missed or treated less than optimally. Marginal impaction detected either in preoperative or intraoperatively imaging studies should be anatomically reduced using the femoral head as a template and augmenting the defect with bone graft if needed. The impacted segment articular surface looks enface during surgery, which is the classic description of such injuries. In the present report, we describe an unusual pattern of marginal impaction injuries appearance in two patients, in which the impacted fragment articular surface is facing toward the joint cavity, which is the reverse of the classic description, alluding to the probable mechanism of its occurrence, the technique for reduction, and the consequences of missing such injuries. Marginal impaction injuries should be diagnosed and treated correctly to preserve joint congruency; however, the surgeon should be aware of the possibility of an unusual pattern of marginal impaction in which the fragment could be reversed, and keeping this possibility in mind would make its diagnosis and management easier.

## Introduction

An isolated posterior wall fracture (elementary type according to the Letournel-Judet system) is one of the most commonly occurring types, representing ∼ 20 to 35% of acetabular fractures. 1 2 3 However, this fracture pattern is not as simple to treat as it seems, with variable prognosis and outcomes, which could be influenced by various factors, including the characteristics of the fracture. 4 5

One of these characteristics is a concomitant “marginal impaction” injury, which, if missed, could lead to less than optimal outcomes. 5 6 Judet et al. 3 described this injury as a rotated impacted fracture segment with depression of osteochondral fragments into the underlying cancellous bone, which occurs in conjunction with a pure posterior fracture-dislocation or as part of complex acetabular fractures and in ∼ 30% of posterior wall fractures. 1 5

In the present case report, we describe two cases presented with an unusual (reversed) pattern of marginal impaction injury associated with acetabular fractures, alluding to the possible explanations of its occurrence, diagnosis, and management.

## Case Reports

The present article was part of a study that was approved by the ethical committee of our institution (IRB no.: 17200019). Informed verbal and written consent was obtained from the patients to use their clinical data and images to publish the present case report.

Two patients, 43 (patient-A) and 19 (patient-B) years old, presented after a motor car accident. After stabilization of their general condition, the initial plain anteroposterior (AP) pelvis radiograph showed fracture-dislocation of the hip in both patients, who were treated by closed reduction under anesthesia and traction. Secondary surveys to investigate acetabular injuries (plain radiograph [AP and Judet views] and pelvis computed tomography [CT] scan). Patient-A had a transverse type with posterior wall fracture, and patient-B had a posterior column fracture. A marginal impaction injury was detected in the CT scan in both patients.

## Surgical Details and Injury Description

**Fig. 1:**
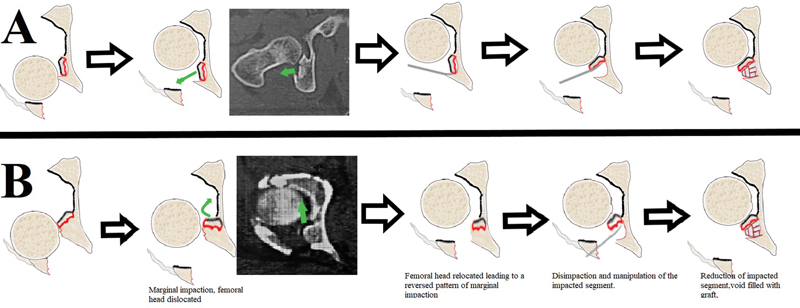
A schematic diagram showing the differences between, ( **A** ) the commonly described marginal impaction pattern, and ( **B** ) the unusual pattern we described in the current report.

**Fig. 2:**
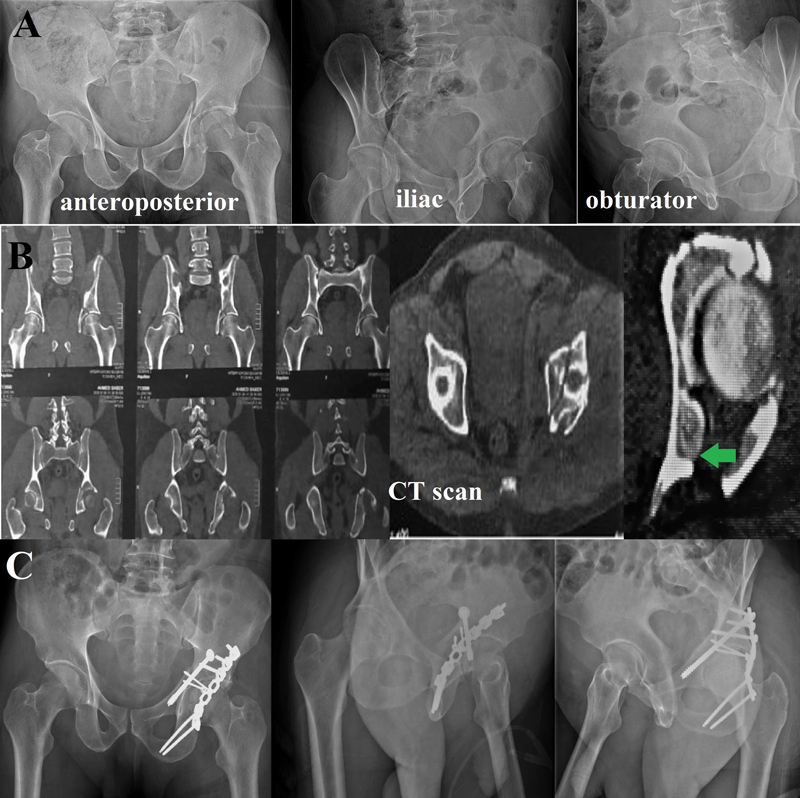
Patient A: Diagnosed as transverse type acetabulum fracture with posterior wall fracture. ( **A and B** ) Preoperative plain radiographs and computed tomography scans (green arrowhead points to the impacted segment). ( **C** ) Postoperative pelvis plain radiographs.

**Fig. 3:**
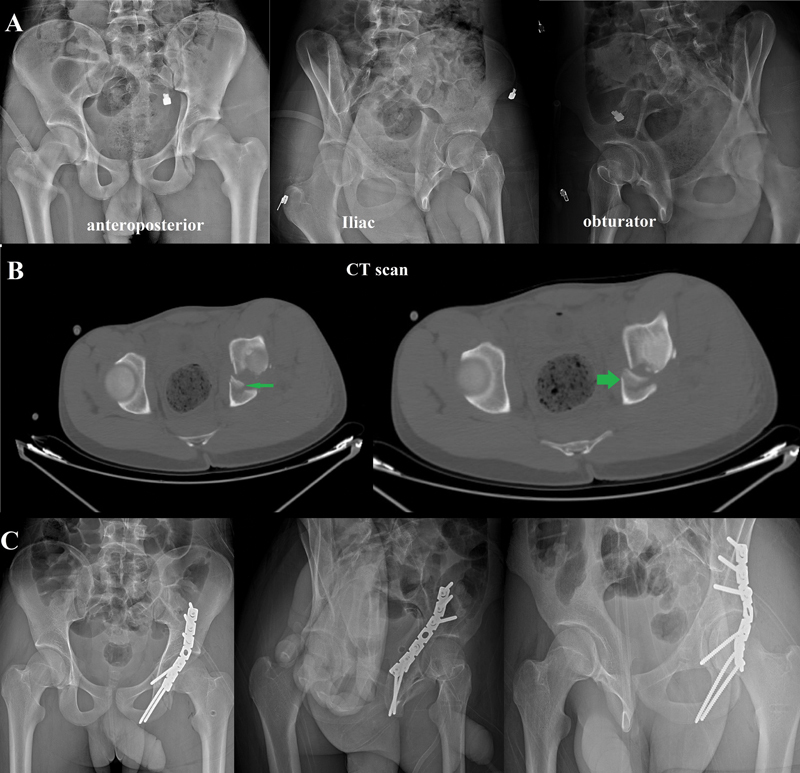
Patient B: Diagnosed as a posterior column fracture. ( **A and B** ) Preoperative plain radiographs and computed tomography scans (green arrowhead points to the impacted segment). ( **C** ) Postoperative pelvis plain radiographs.

Open reduction and internal fixation through the Kocher-Langenbeck approach were performed intraoperatively after joint distraction; we noticed that the orientation of the impacted fragment is reversed compared with the usual marginal impaction appearance, meaning that, instead of having the articular surface of the impacted fragment presented enface (the surgeon having a clear look at the shining articular cartilage of the impacted segment [ Fig. 1A ]), it seemed reversed (the articular surface is looking toward the joint cavity hinged on its inner attachment to the acetabular articular cartilage and the surgeon is looking at the cancellous surface of the impacted segment [ Fig. 1B ]). After re-evaluating the preoperative CT of both patients, we found that the marginal osteochondral impacted fragment is looking toward the acetabulum articular cavity instead of the classic appearance description ( Figs. 2 and 3 ).

The impacted fragment was first disimpacted using an osteotome; then, it was manipulated to its bed, keeping its inner hinge as intact as possible and using the femoral head as a template to ensure perfect reduction. Autogenous bone graft from the greater trochanter was used to support the reduced fragment and to fill the void created by the impaction. Lastly, reduction of the main fracture element and fixation using plate and screws were performed ( Fig. 2 and 3 ).

## Postoperative Protocol

Immediate postoperative plain radiograph series (AP and Judet views) were taken to assess the quality of fracture reduction and the position of the implants. Patients were allowed to mobilize using two axillary crutches with strict nonweight bearing on the operated side. At 6 weeks and after obtaining a follow-up radiograph, the patients were allowed to start partial weight-bearing.

## Discussion

The aims of surgical management of acetabular fractures are mainly to obtain anatomical reduction and to restore the articular congruity for the sake of proper outcomes and to reduce the chance of complications, since fracture malreduction creates articular incongruity leading to hip joint instability and early arthritis. 2 5 6 7

Although posterior wall fractures could be complicated by the presence of comminution or an associated osteochondral marginal impaction, which usually needs disimpaction that might create a void that should be filled by a bone graft, failure to define associated injuries and to manage them properly precipitates less than optimal outcomes. 4 5 6 7

Specific preoperative radiographic signs, such as the seagull sign, help diagnosing a marginal impaction injury; however, in a study by Souza et al., 1 the authors reported 36 patients diagnosed intraoperatively with a marginal impaction injury; however, none of the preoperative radiological reports of these patients mentioned such injury, indicating that this finding could be easily missed in the routine plain radiograph evaluation. 1 Hence, it is essential to evaluate the preoperative CT scan and to confirm the findings intraoperatively. 1 8

The findings of the present report stressed the importance of carefully evaluating the preoperative CT images for the possibility of an unusual pattern like what we have described so that the surgeon would anticipate the technique for its reduction and fixation.

**Fig. 4:**
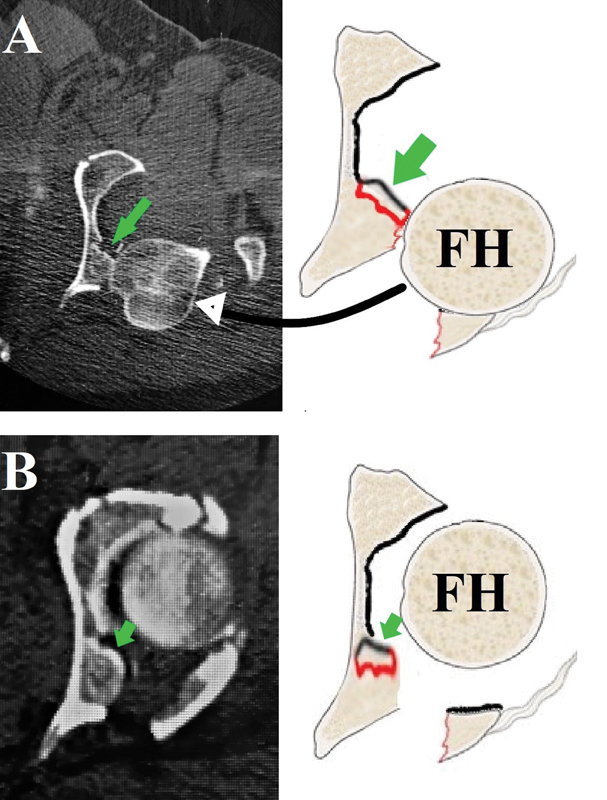
Schematic diagram and computed tomography scan images showing the probable mechanism of injury leading to this unusual pattern of marginal impaction injury. ( **A** ) The head is dislocated, which pushes the marginal impaction segment (green arrowhead) while it is being relocated. ( **B** ) The final picture after relocating the femoral head with the articular surface of the marginally impacted segment facing the joint cavity.

Patient-A was transferred to our hospital after failed closed reduction attempts in another center; the patient presented a CT scan while the hip was dislocated. Based on the appearance of the injury in the CT scan, we hypothesized that this pattern could occur while reducing the hip since the femoral head could hit the outer edge of the impacted fragment, leading to its elevation from its bed and with further femoral head reduction into the joint cavity leading to the flipping of the fragment to be located toward the joint cavity ( Fig. 4 ).

Optimal identification, reduction, and stabilization of marginal impaction remain a vital surgical intervention component. Several studies have demonstrated that articular step-offs are poorly tolerated in acetabular fracture surgery. 9

Hence, the importance of detecting such an unusual pattern lies in first: the surgeon might consider this a loose fragment that, if removed, could affect the joint articular surface. Second: if the surgeon suspects such injury, careful joint distraction is needed to help in reducing this fragment to its bed with caution to preserve the inner hinge where the fragment is attached to the articular cartilage. Third: if this injury was missed intraoperatively, the surgeon might malreduce the posterior wall fracture into the bed of the impacted fragment, leading to joint incongruity and volume reduction.

Marginal impaction injuries should be diagnosed correctly in the preoperative imaging studies; proper reduction would help preserve the joint congruency, leading to acceptable outcomes. The surgeon should be aware of an unusual pattern of marginal impaction where the fragment could be reversed; keeping this possibility in mind would make its diagnosis and management easier.
