# Supplementary material for: Unusual Pattern of Osteochondral Marginal Impaction in Acetabular Fractures: A Report of Two Cases
Source: Rev Bras Ortop (Sao Paulo). 2022 Sep 5;59(Suppl 1):e83–7. doi: 10.1055/s-0042-1755347 (PMC11254432; doi:10.1055/s-0042-1755347)
Supplement: Related texts

# Research article: Padrão incomum de impacto marginal osteocondral em fraturas acetabulares: Um relatório de dois casos

**Keywords:** acetábulo, fraturas do quadril, fraturas ósseas, osteonecrose

## Abstract

As fraturas acetabulares podem ser acompanhadas por lesões de impacto articular, afetando os desfechos se não vistas ou tratadas de modo incompleto. A impactação marginal detectada em estudos de imagem pré- ou intraoperatórios deve ser reduzida anatomicamente usando a cabeça femoral como modelo e aumentando o defeito com enxerto ósseo, se necessário. A superfície articular do segmento impactado parece voltada para a frente durante a cirurgia, que é a descrição clássica de tais lesões. No relatório atual, descrevemos um padrão incomum de aparecimento de lesões de impacto marginal em dois pacientes, onde a superfície articular do fragmento impactado está voltado para a cavidade articular, sendo o inverso da descrição clássica, aludindo ao mecanismo provável de sua ocorrência, à técnica de redução e às consequências de ignorar tal lesão. Lesões de impacto marginal devem ser diagnosticadas e tratadas corretamente para preservar a congruência articular; no entanto, o cirurgião deve estar ciente da possibilidade de um padrão incomum de impacto marginal no qual o fragmento poderia ser revertido; manter esta possibilidade em mente facilitaria seu diagnóstico e manejo.

## Introdução

Uma fratura isolada da parede posterior (tipo elementar de acordo com o sistema Letournel-Judet) é um dos tipos que ocorrem mais comumente, representando ∼ 20 a 35% das fraturas acetabulares. 1 2 3 No entanto, este padrão de fratura não é tão simples de tratar como parece, com prognóstico e desfechos variáveis que podem ser influenciados por diversos fatores, incluindo características da fratura. 4 5

Uma destas características é uma lesão concomitante de "impacto marginal" que, se não identificada, poderia levar a resultados menos que ideais. 5 6 Judet et al. 3 descreveram esta lesão como um segmento de fratura impactado rotativo com depressão de fragmentos osteocondrais no osso esponjoso subjacente que ocorre em conjunto com uma fratura-luxação posterior pura ou como parte de fraturas acetabulares complexas e em ∼ 30% das fraturas posteriores da parede. 1 5

Descrevemos aqui dois casos apresentados com um padrão incomum (invertido) de lesão de impacto marginal associada a fraturas acetabulares, aludindo às possíveis explicações de sua ocorrência, diagnóstico e manejo.

## Relatos de casos

O presente artigo fez parte de um estudo aprovado pelo comitê de ética da nossa instituição (IRB no.: 17200019). Os pacientes cederam consentimento informado verbal e por escrito para o uso de seus dados clínicos e imagens na publicação do presente relato de caso.

Dois pacientes, de 43 (paciente-A) e 19 (paciente-B) anos, apresentaram-se após acidente automobilístico. Após a estabilização da condição geral, a radiografia posteroanterior (PA) inicial da pelve mostrou fratura-luxação do quadril em ambos os pacientes, que foram tratados por redução fechada sob anestesia e tração. Houve investigações secundárias para investigar lesões acetabulares (radiografia simples [PA e Judet] e tomografia computadorizada [TC] da pelve). O paciente-A tinha um tipo transverso com fratura posterior da parede e o paciente-B tinha uma fratura posterior da coluna. Foi detectada uma lesão de impacto marginal na TC em ambos os pacientes.

## Detalhes cirúrgicos e descrição da lesão

A redução aberta e a fixação interna por meio da abordagem Kocher-Langenbeck foram realizadas no intraoperatório após distração articular; notamos que a orientação do fragmento impactado é invertida em comparação com a aparência usual de impacto marginal, o que significa que, em vez de ter a superfície articular do fragmento impactado se apresentando frontalmente (o cirurgião tendo uma visão clara da cartilagem articular brilhante do segmento impactado [ Fig. 1A ]), ela parecia invertida (a superfície articular está virada para a cavidade articular presa na sua conexão interna à cartilagem articular acetabular e o cirurgião está olhando para a superfície esponjosa do segmento impactado [ Fig. 1B ]). Após reavaliar a tomografia pré-operatória de ambos os pacientes, descobrimos que o fragmento impactado osteocondral marginal está virado para a cavidade articular do acetábulo em vez de em acordo com a descrição clássica da aparência ( Figs. 2 e 3 ).

**Fig. 1:** Diagrama esquemático mostrando as diferenças entre, ( **A** ) o padrão de impacto marginal comumente descrito e ( **B** ) o padrão incomum descrito no relatório atual.

**Fig. 2:** Paciente A: diagnosticado como fratura de acetábulo tipo transverso com fratura posterior da parede. ( **A** e **B** ) Radiografias simples pré-operatórias e tomografia computadorizada (ponta de seta verde aponta para o segmento impactado). ( **C** ) Radiografias simples da pelve pós-operatória.

**Fig. 3:** Paciente B: diagnosticado como fratura posterior da coluna. ( **A** e **B** ) Radiografias simples pré-operatórias e tomografia computadorizada (ponta de seta verde aponta para o segmento impactado). ( **C** ) Radiografias pós-operatória simples da pelve.

O fragmento impactado foi primeiro desimpactado usando um osteótomo; depois, foi manipulado para a sua cama, mantendo sua dobradiça interna o mais intacta possível e usando a cabeça femoral como modelo para garantir uma redução perfeita. Usou-se enxerto ósseo autógeno do maior trocânter para apoiar o fragmento reduzido e preencher o vazio criado pela impactação. Por fim, fez-se a redução do elemento principal da fratura e a fixação utilizando placa e parafusos ( Figs. 2 e 3 ).

## Protocolo pós-operatório

Séries de radiografia simples pós-operatórias imediatas (PA e Judet) foram feitas para avaliar a qualidade da redução da fratura e a posição dos implantes. Os pacientes foram autorizados a se mobilizar usando duas muletas axilares com deambulação rigorosamente sem peso no lado operado. Às 6 semanas e após a obtenção da radiografia de seguimento, os pacientes foram autorizados a iniciar a deambulação com apoio parcial do peso.

## Discussão

Os objetivos do manejo cirúrgico das fraturas acetabulares são principalmente obter redução anatômica e restaurar a congruência articular para efeitos adequados e redução da chance de complicações, uma vez que a má redução da fratura cria incongruência articular, levando a instabilidade articular e artrite precoce. 2 5 6 7

Embora as fraturas posteriores da parede possam ser complicadas pela presença de cominução ou impactação marginal osteocondral associada, elas geralmente precisam de desimpactação, o que pode criar um vazio que deve ser preenchido por um enxerto ósseo. Deixar de definir lesões associadas e gerenciá-las adequadamente gera resultados longe dos ideais. 4 5 6 7

Há sinais radiográficos pré-operatórios específicos para diagnosticar uma lesão de impacto marginal, como o sinal de gaivota. No entanto, um estudo de Martins e Souza et al. 1 relatou 36 pacientes diagnosticados intraoperatoriamente com lesão de impacto marginal; nenhum dos relatos radiológicos pré-operatórios destes pacientes mencionou tal lesão, indicando que tal achado poderia ser facilmente perdido na avaliação de radiografia simples de rotina. 1 Por isso, é essencial avaliar a tomografia pré-operatória e confirmar os achados intraoperatoriamente. 1 8

Os achados do atual relato enfatizaram a importância de avaliar cuidadosamente as imagens de tomografias pré-operatórias para a possibilidade de um padrão incomum como o que descrevemos para que o cirurgião antecipasse a técnica para sua redução e fixação.

O paciente-A foi transferido para o nosso hospital após tentativas fracassadas de redução fechada em outro centro; o paciente apresentou uma tomografia enquanto o quadril estava deslocado. Com base no aparecimento da lesão na TC, supomos que este padrão poderia ocorrer enquanto se reduzia o quadril, pois a cabeça femoral poderia atingir a borda externa do fragmento impactado, levando à elevação do seu leito e a uma maior redução da cabeça femoral na cavidade articular, com a inversão do fragmento a ser localizado em direção à cavidade articular ( Fig. 4 ).

**Fig. 4:** Imagens de diagrama esquemático e tomografia computadorizada mostrando o provável mecanismo de lesão que leva a este padrão incomum de lesão de impacto marginal. ( **A** ) A cabeça está deslocada, o que empurra o segmento de impacto marginal (ponta de seta verde) enquanto está sendo realocado. ( **B** ) Quadro final após a realocação da cabeça femoral com a superfície articular do segmento marginalmente impactado frente à cavidade articular.

A identificação, a redução e a estabilização da impactação marginal permanecem componentes vitais de intervenção cirúrgica. Vários estudos demonstraram que os *step-offs* articulares são mal tolerados na cirurgia de fratura acetabular. 9

Assim, a importância de detectar um padrão tão incomum está em primeiro lugar: o cirurgião pode considerar este um fragmento solto que, se removido, pode afetar a superfície articular da articulação. Segundo: se o cirurgião suspeitar de tal lesão, é necessária uma cuidadosa distração articular para ajudar na redução desse fragmento ao seu leito com cautela para preservar a dobradiça interna onde o fragmento está preso à cartilagem articular. Terceiro: se essa lesão não for detectada no intraoperatório, o cirurgião pode reduzir incorretamente a fratura da parede posterior no leito do fragmento impactado, levando à incongruência articular e redução de volume.

Lesões de impacto marginal devem ser diagnosticadas corretamente nos estudos de imagem pré-operatória; a redução adequada ajudaria a preservar a congruência conjunta que leva a resultados aceitáveis. O cirurgião deve estar ciente de um padrão incomum de impacto marginal no qual o fragmento possa ser invertido; manter tal possibilidade em mente facilitaria seu diagnóstico e gestão.
